# Exploring hub genes and crucial pathways linked to oxidative stress in bipolar disorder depressive episodes through bioinformatics analysis

**DOI:** 10.3389/fpsyt.2024.1323527

**Published:** 2024-03-06

**Authors:** Shasha Wu, Haiyang Hu, Yilin Li, Yan Ren

**Affiliations:** ^1^ Department of Psychiatry, Shanxi Bethune Hospital, Shanxi Academy of Medical Sciences, Tongji Shanxi Hospital, Third Hospital of Shanxi Medical University, Taiyuan, China; ^2^ Tongji Hospital, Tongji Medical College, Huazhong University of Science and Technology, Wuhan, China; ^3^ Third Hospital of Shanxi Medical University, Shanxi Bethune Hospital, Shanxi Academy of Medical Sciences, Tongji Shanxi Hospital, Taiyuan, China

**Keywords:** bipolar disorder, bipolar depression, oxidative stress, hub gene, diagnostic, bioinformatics

## Abstract

**Background:**

Bipolar disorder (BD) is a complex and serious psychiatric condition primarily characterized by bipolar depression, with the underlying genetic determinants yet to be elucidated. There is a substantial body of literature linking psychiatric disorders, including BD, to oxidative stress (OS). Consequently, this study aims to assess the relationship between BD and OS by identifying key hub genes implicated in OS pathways.

**Methods:**

We acquired gene microarray data from GSE5392 through the Gene Expression Omnibus (GEO). Our approach encompassed differential expression analysis, weighted gene co-expression network analysis (WGCNA), and Protein-Protein Interaction (PPI) Network analysis to pinpoint hub genes associated with BD. Subsequently, we utilized Gene Ontology (GO), Kyoto Encyclopedia of Genes and Genomes (KEGG), and Gene Set Enrichment Analysis (GSEA) to identify hub genes relevant to OS. To evaluate the diagnostic accuracy of these hub genes, we performed receiver operating characteristic curve (ROC) analysis on both GSE5388 and GSE5389 datasets. Furthermore, we conducted a study involving ten BD patients and ten healthy controls (HCs) who met the special criteria, assessing the expression levels of these hub genes in their peripheral blood mononuclear cells (PBMCs).

**Results:**

We identified 411 down-regulated genes and 69 up-regulated genes for further scrutiny. Through WGCNA, we obtained 22 co-expression modules, with the sienna3 module displaying the strongest association with BD. By integrating differential analysis with genes linked to OS, we identified 44 common genes. Subsequent PPI Network and WGCNA analyses confirmed three hub genes as potential biomarkers for BD. Functional enrichment pathway analysis revealed their involvement in neuronal signal transduction, oxidative phosphorylation, and metabolic obstacle pathways. Using the Cytoscape plugin “ClueGo assay,” we determined that a majority of these targets regulate neuronal synaptic plasticity. ROC curve analysis underscored the excellent diagnostic value of these three hub genes. Quantitative reverse transcription-PCR (RT-qPCR) results indicated significant changes in the expression of these hub genes in the PBMCs of BD patients compared to HCs.

**Conclusion:**

We identified three hub genes (TAC1, MAP2K1, and MAP2K4) in BD associated with OS, potentially influencing the diagnosis and treatment of BD. Based on the GEO database, our study provides novel insights into the relationship between BD and OS, offering promising therapeutic targets.

## Introduction

Bipolar disorder, a chronic and recurrent condition marked by mood state and energy fluctuation, can be categorized into BD type I (BD-I) and BD type II (BD-II), based on the severity of elevated moods (1). BD-I is characterized by manic episodes, while BD-II presents with mild manic and major depressive episodes (2). BD has a high relapse rate, a high incidence of other comorbidities, poor treatment adherence, and only 20% of patients with bipolar disorder experiencing depressive episodes are diagnosed within a year of seeking treatment, severely affecting patients’ quality of life and social functioning (3). However, the underlying pathogenesis of BD remains elusive. With the rapid development of high-throughput sequencing technology and bioinformatics has significantly improved the diagnosis and treatment of diseases (4). However, there is a lack of sensitive and specific biomarkers that can be widely used in the clinical diagnosis of BD (5). Thus, early intervention and the search for validated biomarkers are imperative for comprehending the course and treatment of BD (6).

Currently, it is widely acknowledged that BD’s pathogenesis arises from a complex interplay of genetic and environmental factors (7). These factors encompass processes involving neuronal-glial plasticity, monoaminergic signaling, inflammatory homeostasis, cellular metabolic pathways, and mitochondrial dysfunction (8). Neurotransmitter imbalances, oxidative stress, and genetic factors are among the key contributors to BD’s pathophysiology (9). A growing body of evidence underscores the pivotal role of oxidative stress in BD’s pathophysiology (10). The imbalance between antioxidant and oxidative processes results in the production of free radicals and reactive oxygen species (ROS), leading to damage to critical macromolecules such as DNA, proteins, and lipids (11). Consequently, this disruption reduces metabolism and triggers neuroplastic dysfunction, further impairing brain function (12). Studies have demonstrated significantly elevated levels of lipid peroxidation, natural nitric oxide, and DNA and RNA damage in BD patients compared to HCs, reinforcing the potential involvement of OS in the pathogenesis of BD (13). Moreover, BD patients exhibit a decrease in total antioxidant capacity following their first manic episode, with anti-oxidant mechanisms remaining stable regardless of the number of manic episodes, but increasing in the later stages of mania (14). OS also exerts adverse effects on calcium levels, membrane permeability, and mitochondrial defense systems (15). Mitochondrial dysfunction escalates ROS production, exacerbating OS, and has emerged as a critical factor in BD (16). Thus, OS significantly correlated with BD’s pathophysiology and remains a subject of keen interest in psychiatric disorder research.

Given that depressive episodes outweigh manic states in the course of BD and possess distinct pathological mechanisms, this study focuses on bipolar depression, aiming to uncover the relationship between OS and BD in terms of genes and pathways associated with OS (17). Bioinformatics-based gene expression profiling provides an important and effective tool for diagnosing biomarkers for various diseases. Weighted Gene Co-expression Network Analysis (WGCNA), as a bioinformatics tool for analyzing gene expression patterns of multiple samples, clusters and forms modules of genes by similar gene expression patterns and analyze the relationship between modules and specific features to establish the relationship between phenotypic traits and gene expression data (18). In investigate the molecular and genetic mechanisms between BD and OS, a comprehensive network of OS-related differentially expressed genes (OS-DEGs) was first constructed through a bioinformatics-based gene expression profiling approach. Three hub genes linked to OS and BD were subsequently identified through the Protein-Protein Interaction (PPI) Network and WGCNA. Furthermore, biological processes and pathways were explored using Gene Ontology (GO) and Gene Set Enrichment Analysis (GSEA). Logistic regression analyses were employed to establish a BD diagnostic model, which was subsequently validated in two additional datasets, GSE5388 and GSE5389, comprising post-mortem brain tissues from BD patients. The area under the curve (AUC) values indicate that the model exhibits superior diagnostic performance, holding promise for BD diagnosis. Finally, the expression levels of these hub genes were assessed in peripheral blood mononuclear cells (PBMCs). In summary, by identifying the expression of multiple genes in the blood, the model is expected to guide the diagnosis of BD patients. This study will not only deepen our understanding of pathogenesis of BD, but also provide a practical framework to unravel the molecular mechanisms underpinning BD’s pathogenesis at the systems biology level, and offer some new ideas for its diagnosis and treatment.

## Materials and methods

### Subject recruitment

This study was approved by the medical ethics committee of Shanxi Bethune Hospital, with the Approval Notice Number: YXLL-2020-001. Prior to enrollment in our study, all participants provided written informed consent, and for teenage participants, consent was obtained from their parents. Every procedure adhered to the applicable rules and regulations. To ensure the uniformity of the BD subtype within the patient group, inclusion criteria mandated that patients be diagnosed with BD-II. The diagnosis of BD patients experiencing depressive episodes was established using the Diagnostic and Statistical Manual of Mental Disorders (5^th^ edition), the 24-item Hamilton Depression Rating Scale (HDRS), and the Young Mania Rating Scale (YMRS). Specifically, patients with BD in a manic state were identified with a YMRS score > 6 and a 24-item HDRS score ≥20. Moreover, the diagnosis was independently confirmed by at least two experienced and licensed psychiatrists. Exclusion criteria encompassed individuals with any concurrent physical or psychological conditions, as well as those grappling with substance-related issues. Meanwhile, ten healthy individuals with no family history of psychiatric conditions were selected as the control group. Exclusion criteria for all participants included acute or persistent infections, autoimmune disorders or mental disorders, pregnancy, lactation, or menstruation. Comprehensive participant details are presented in **Table 1**.

**Table 1:** Demographic and clinical details of recruited subjects.

Variables	BD	HC	*p-value* ^a^
Sample size	10	10	–
Age (year)^b^	20.50±11.02	26.30±4.40	0.158
BMI (kg/m²)^b^	23.43±5.14	22.80±4.14	0.765
Gender (M/F)	4/6	5/5	1.00
**Dignosis**			
BD typle I	2(20%)	–	–
BD typle II	6(60%)	–	–
BD uncerten	2(20%)	–	–
**Clinical charactors**			
CGI score^b^	4.40±0.97	–	–
Age of first onset (age)^c^	15.00(13, 16.75)	–	–
Total course of disease (month)^b^	28.70±21.05	–	–
Dignostic age (year)^b^	16.00(14.00, 18.00)	–	–
Medication (Yes/No)	(10/0)	–	–
**HAMD factor score**			
Anxiety/Somatization^b^	5.60±3.60	–	–
Weight^b^	0.50(0.00,2.00)	–	–
Cognitive impairment	3.00(1.50,4.25)	–	–
Diurnal variation^b^	0.00(0.00,0.50)	–	–
Slowness^b^	5.30±2.67	–	–
Sleep disorder^b^	2.40±2.17	–	–
Despair sense^b^	3.30±2.11	–	–
Suicide^c^	2.00(1.75,2.25)	–	–
HAMD-24 total score^b^	23.20±11.77		
YMRS scores^b^	12.30±3.09		
**HAMA factor score**		–	–
Somatic anxiety^b^	4.70±3.68	–	–
Mental anxiety^b^	7.50±3.03	–	–
HAMA-14 total score^b^	12.20±6.39		
**Medicine**		–	–
Lithium	8(80%)	–	–
Lamotrigine	1(10%)	–	–
Sodium valproate	4(40%)	–	–
Antipsychotics	6(60%)	–	–
Antidepressants	10(10%)	–	–

BD, bipolar disorder; HC, health control; M/F, male/female; BMI, Body Mass Index; HAMD, Hamilton Depression Scale; HAMA, Hamilton Anxiety Scale; YMRS, Young Mania Rating Scale.

a two independent samples t-test for continuous variables (age and BMI); Fisher's exact test for categorical variables (sex).

b Values expressed as means ± SDs.

c Values expressed as median (int erquartile range, IQR).

### Microarray expression data acquisition and processing

Three microarray expression data of BD and their clinical details were obtained from the GEO (https://www.ncbi.nlm.nih.gov/geo/) databases (GSE5392, GSE5388, GSE5389). GSE5392 contained post-mortem brain tissue (dorsolateral prefrontal cortex and orbitofrontal cortex) from 40 BD patients and matched 42 HCs. Subsequently, GSE5392 combined the expression matrix with the platform of GPL96 ([HG-U133A] Affymetrix Human Genome U133A Array), which allowed the probes to be transformed into matching gene symbols. When a gene has multiple probe expression data, the median expression value was calculated as the gene expression level. These sample data were then normalized by quantile normalization. Samples from two datasets GSE5388 and GSE5389 were defined as verification cohort after integrating. GSE5388 annotated by GPL96 included 30 BD patients and 30 HCs, and GSE5389 annotated by GPL96 included 10 BD patients and 11 HCs. In addition, the same data processing was required for GSE5388 and GSE5389.

### Oxidative stress-related genes

The Genecard website (https://www.genecards.org/) was used to find OS-related genes. Targets with a relevance score exceeding 7 were included and we obtained 824 genes (**Supplementary Table S1**).

### Differential expression analysis

To conduct differential expression analyses between BD patients and healthy controls (HCs), we utilized the R packages “limma” and “impute”. Differentially expressed genes (DEGs) were identified using a significance threshold of *P-value* < 0.05 and a 1.2-fold change cutoff for both up-regulation and down-regulated genes. The DEGs were visually represented through volcano plots and heat maps using pheatmap and ggplot2 packets of R language.

### Weighted gene co-expression network analysis

First, we calculated the median absolute deviation (MAD) of each gene using gene expression profiles, and excluded the top 50 percent of genes with the smallest MAD. The GoodSamplesGenes method of R package WGCNA was used to remove the outlier genes and samples, and the gene co-expression network was further generated to explore the relationship between co-expressed genes and phenotypes (18). Pearson’s correlation matrix and an average linkage approach were employed for all pairwise gene comparisons. Subsequently, a scale-free co-expression network was created utilizing a weighted adjacency matrix and a soft-thresholding parameter β, which β was a soft threshold parameter that emphasizes strong correlations between genes and ignores weak correlations (19). The adjacency matrix transformed a topological overlap matrix (TOM) with a selected power of 14, which measured the net connectivity of a gene, defined as the sum of the gene’s adjacencies to all other genes in the net, and the corresponding dissimilarity (1-TOM) was calculated. Cluster analysis was then utilized to identify correlations within each module. Simultaneously, average linkage hierarchical clustering was applied using the TOM-based dissimilarity measure, with a minimum gene group size of 30, facilitating the arrangement of genes with similar expression patterns. In addition, we merged the modules with distance less than 0.25.

### Construction of protein-protein interaction network and hub genes selection

STRING (http://string-db.org/) serves as an invaluable online database for analyzing functional protein interaction networks (20). We entered the screened DEGs associated with OS into the STRING database. Subsequently, we extracted all PPI pairs with a combined score exceeding 0.4. High-degree nodes that played a pivotal role in maintaining network stability were identified. The top 10 genes based on their degree values were employed to identify the network’s hub genes with the assistance of Cytoscape’s additional plugins, CytoNCA and CytoHubba. Cytoscape software (version 3.9.0) was utilized for calculating the degree of each node (21). Organizing these node scores allowed us to pinpoint significant nodes within the protein interaction network. The identified hub genes were associated with OS. Hub genes were associated with OS, the DEGs in BD patients were screened with genes obtained from WGCNA and the top 10 highest degree values genes from PPI Network were taken to screen the intersection genes using the “Venn Diagram” package in R software. Differences in hub gene expression between BD and HCs were depicted using violin plots.

### A functional enrichment pathway’s analysis

To elucidate the molecular pathways influencing OS-DEGs in BD, we GO and KEGG enrichment analyses. The GO terms encompass three categories: biological process (BP), cellular component (CC), and molecular function (MF). Gene functions were systematically analyzed, annotated, and visualized through the KEGG database. Additionally, we harnessed the comprehensive functional annotation tool DAVID (http://david.abcc.ncifcrf.gov/) to probe the functional significance of OS-DEGs, given its capacity to discern the biological implications of a multitude of genes (22). The GO enrichment and KEGG pathway analyses were conducted using R’s cluster Profiler package. We proceeded to analyze molecular profiles and identify vital pathways or sets of biologically related genes implicated in complex human diseases using GSEA via the Sangerbox tool (http://sangerbox.com/Tool). Specifically, we scrutinized mRNA expression levels between BD patients and HCs. Further enhancing our comprehension of the biological relevance of OS-DEGs and their regulators in BD, we applied functional enrichment analysis with ClueGO, which rendered functionally associated genes in the form of a network and chart with clusters (23).

### Screening and validation of hub genes

In pursuit of early disease detection and diagnosis, the generalized linear regression analytic model of logistic regression emerged as a valuable tool. In our study, we harnessed three GEO databases for logistic regression analysis, dividing the samples into two response factors representing BD patients (with a response variable of 1) and HCs (with a response variable of 0). To assess the diagnostic capabilities of hub genes, we conducted an ROC survival analysis (24). AUC was employed as a concise accuracy index. Typically, markers with higher values exhibit a higher probability of correctly identifying the feature under scrutiny. ROC curves with AUC values less than or equal to 0.5 signify an unreliable model, while values between 0.51 and 0.7 denote poor model accuracy. Models attain moderate accuracy with AUC values between 0.71 and 0.9, while those surpassing 0.9 demonstrate a high degree of accuracy. For these analyses, we employed the statistical package pROC in R software.

### Quantitative reverse transcription-PCR

Venous blood was collected from all participants using blood collection tubes containing ethylenediaminetetraacetic acid (EDTA). Peripheral blood mononuclear cells (PBMCs) were subsequently isolated from whole blood samples using Ficoll solution (Solarbio Life Sciences, China). Total RNA was extracted from the PBMCs employing the TransZol Up Plus RNA Kit (TransGen, China). RNA quality assessment was conducted using a NanoVue Plus spectrophotometer (Biochrom, UK), followed by RT-qPCR on a CFX96 real-time PCR detection system (Bio-Rad, USA). The expression of GADPH, which encodes glyceraldehyde 3-phosphate dehydrogenase, was employed as a reference for data standardization. Please see **Table 2** for a list of the primers used in the RT-qPCR experiment. The 2^-ΔΔCt^ method was employed to calculate the fold changes of the specified genes.

**Table 2:** RT-qPCR primers.

Primers	Sequences (5’-3’)
TAC1	Forward: CGCCCCTCTTTGTCTTCCAC
	Reverse: GAATTTTCTGCCCCACAACGG
MAP2K1	Forward: TCAGACCTGCTCCCAACAGAC
	Reverse: AGTTAGCCAAGATCGCACCA
MAP2K4	Forward: TCACCTGCTAGAACCTCTCGT
	Reverse: ACAAAGCCTAAGGTTCAGTGC
GADPH	Forward: CAACAGCCTCAAGATCATCAGGT
	Reverse: CATACCATGAGTCCTTCCACGAT

### Statistical analysis

R software was used for all analyses. The t-test and Mann-Whitney U-test were selected based on whether the data was normally distributed. All data were analyzed using SPSS 26.0 software, and significance was defined based on normalized *P-values* < 0.05.

## Results

### Identification of DEGs in BD patients

**Figure 1:**
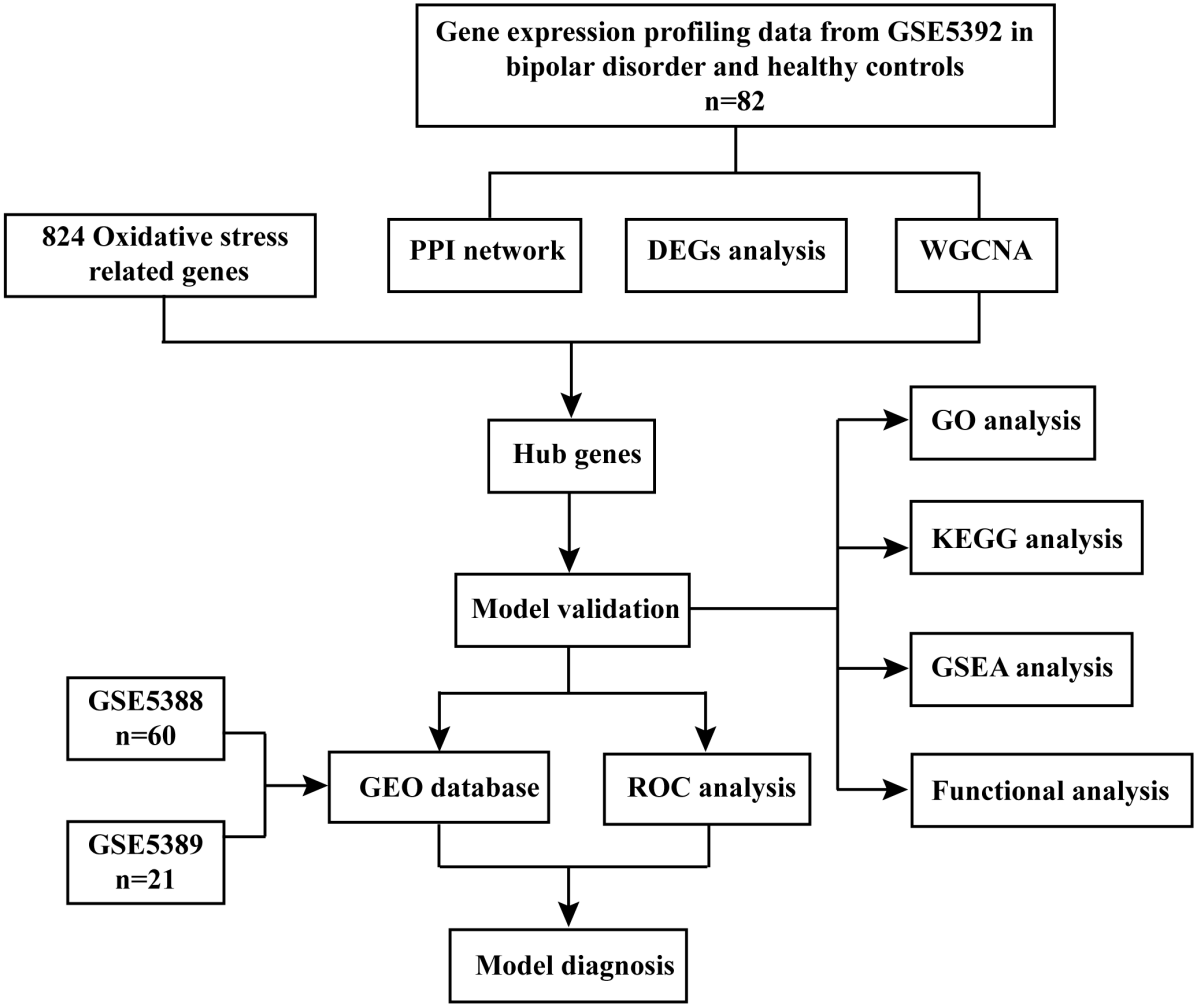
Flow chart of the data analysis process.

**Figure 2:**
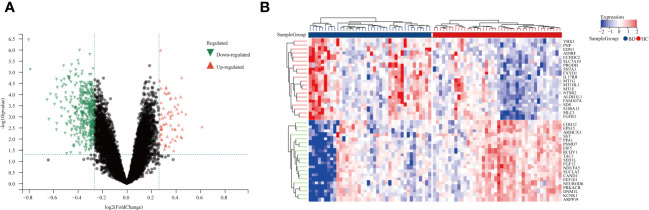
Differentially expression genes between BD patients and HCs. **(A)** A volcano plot of 480 DEGs, Red genes represent up-regulated genes; Green genes represent down-regulated genes; Black genes represent unchanged genes. **(B)** The heatmap shows the top 20 genes significantly highly expressed in BD patients or HCs. Red genes represent higher expression; blue genes represent lower expression.

We studied the high-throughput sequencing data of 82 patients with BD obtained from GEO database. **Figure 1** displays the study’s flowchart and the detail of DEGs expression in **Supplementary Table S2**. To identify genes related to BD, 480 DEGs were successfully identified from GSE5392 with the screening conditions “*P-value* < 0.05 and 1.2-fold change”, including 69 upregulated and 411 downregulated genes. These DEGs were displayed in a plot of a volcano (**Figure 2A**) and listed in **Supplementary Table S3**. The top 20 differentially expressed genes were shown as a heatmap in **Figure 2B**.

### Co-expression network construction by WGCNA

**Figure 3:**
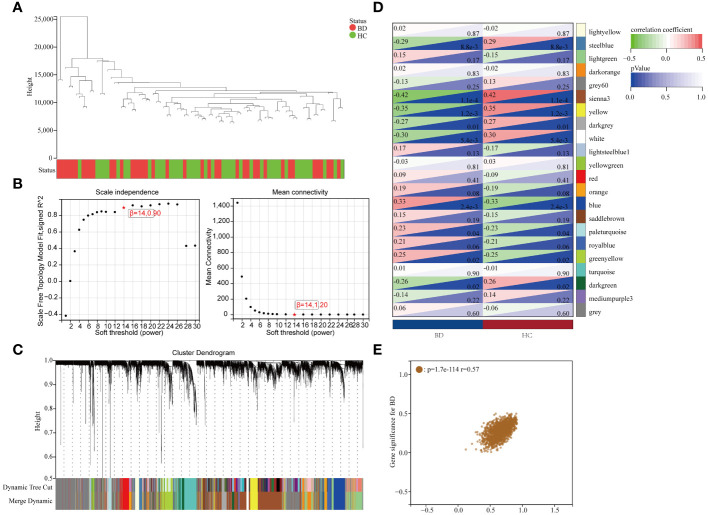
Results of the WGCNA. **(A)** Using a trait heatmap to cluster the dendrogram of samples. **(B)** Determination of the power of soft-thresholding. **(C)** Gene dendrogram with clustering. **(D)** Module-trait relationship heatmap. **(E)** Scatter plot demonstrating the relationship between gene module membership in the sienna3 module and gene relevance.

Upon excluding aberrant samples and applying gene filters, we employed Pearson’s correlation coefficient to perform sample clustering in GSE5392. We extracted expression profiles of 6412 genes from 82 samples in GSE5392, which were then utilized to construct a weighted gene co-expression network (see **Figure 3A**). To transform the adjacency matrix into a topological overlap matrix, the soft-thresholding parameter was set at 14 (achieving a scale-free *R^2 =^* 0.85, as shown in **Figure 3B**). Subsequently, a hierarchical clustering tree was generated following a dynamic hybrid cut (**Figure 3C**), with a minimum module size of 30 and a cut height of 0.25, resulting in the identification of 22 distinct co-expression modules (**Figure 3D**; non-clustering DEGs shown in grey). Notably, the sienna3 module exhibited the highest positive correlation with BD, particularly in relation to clinical characteristics such as the Gleason score (*r* = 0.42, *p* = 1.1e-4; **Figure 3D**). The sienna3 module, housing a total of 218 genes with the largest absolute correlation coefficients, was selected for further analysis. Additionally, correlation analysis between module membership (MM) and gene significance (GS) demonstrated significant associations of these genes with both the module and the phenotype (*r* = 0.57, *p* = 1.7e-114) (**Figure 3E**). The module genes identified in the WGCNA can be found in **Supplementary Table S4**.

### Gene ontology and pathway enrichment analysis of OS-DEGs

**Figure 4:**
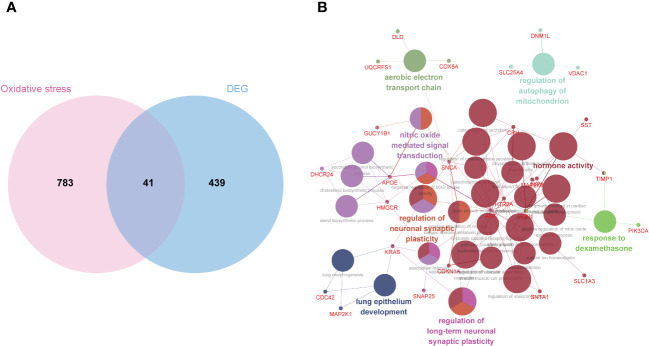
Biological process annotation of OS-DEGs. **(A)** Venn diagram revealed 41 intersect genes in the OS-DEGs. **(B)** Functions and pathways of the OS-related genes were visualized using ClueGO.

To identify OS-DEGs (DEGs related to oxidative stress), we collected a set of 480 DEGs and 824 OS-related genes. A Venn diagram analysis revealed a total of 41 target genes (**Figure 4A**). Previously, researchers have employed ClueGO, a widely-used Cytoscape plugin, to elucidate the biological significance and relationships among various functional groups within biological networks, aiming to uncover the mechanistic actions of these 41 prospective targets. Notably, the results uncovered several functional categories linked to biological processes, including the aerobic electron transport chain, regulation of mitochondrion autophagy, response to dexamethasone, regulation of long-term neuronal synaptic plasticity, lung epithelium development, regulation of neuronal synaptic plasticity, and nitric oxide mediated signal transduction (**Figure 4B**). Detailed findings from the ClueGO analysis are presented in **Supplementary Table S5**.

**Figure 5:**
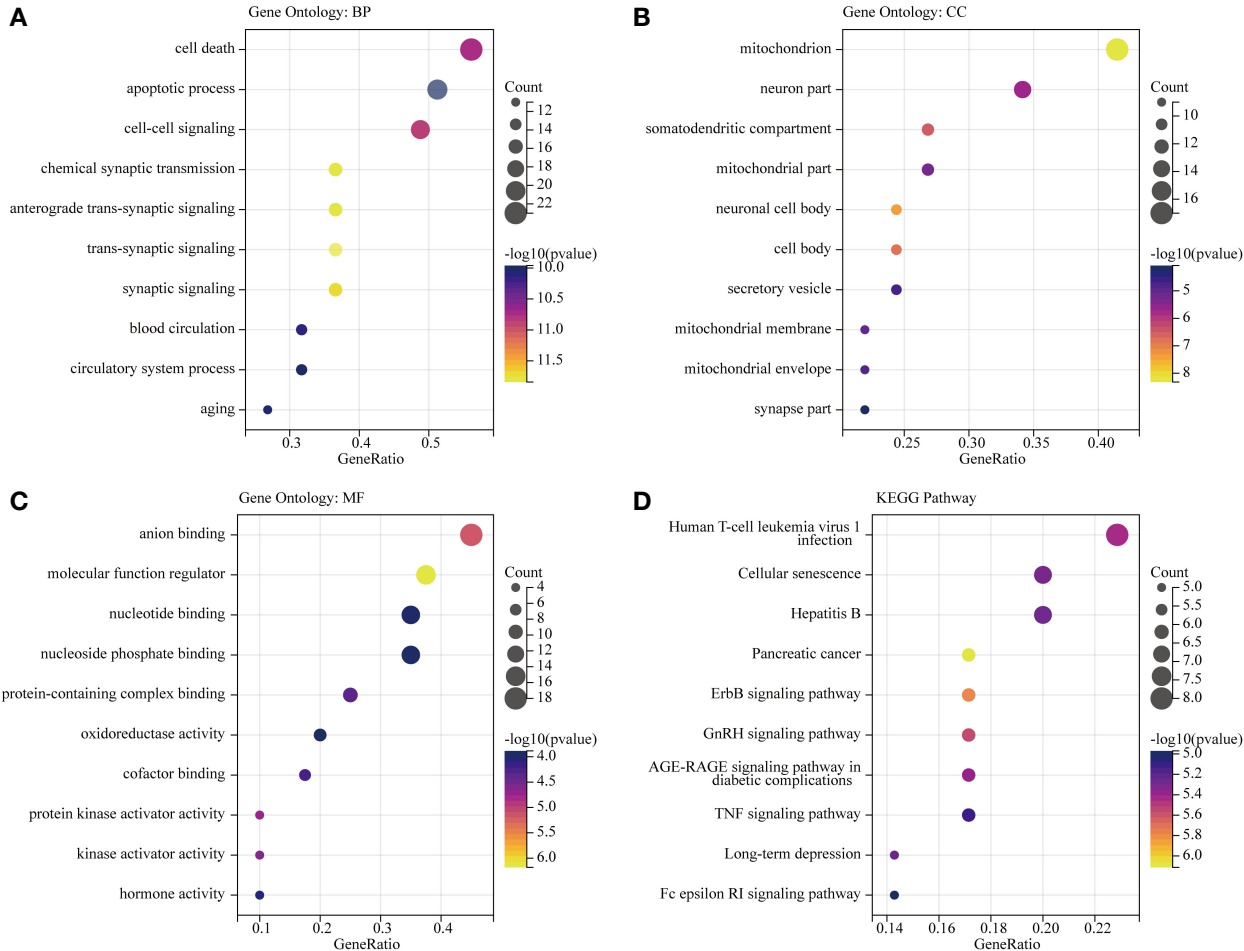
GO functional and KEGG pathway analysis of OS-DEGs. Top 10 enriched GO and KEGG terms of OS-DEGs in BD. The ratio of enriched genes to the 41 target genes is referred to as the gene ratio. Amount of enriched genes is denoted by the gene count. The size of the bubbles is determined by the significant enhanced item’s target concentration. The bubble’s color depth serves as a sign of significance. **(A–C)** GO function analysis of OS-DEGs. **(D)** KEGG pathway analysis of OS-DEGs.

To gain insights into the potential enriched pathways associated with OS-DEGs, we conducted GO and KEGG pathway enrichment analyses. In total, 41 OS-DEGs were implicated in GO enrichment. In terms of biological processes, the GO enrichment analysis revealed that these target genes were primarily associated with trans-synaptic signaling, cell-cell signaling, circulatory system process, and apoptotic processes (**Figure 5A**). Regarding cellular components, the target genes exhibited significant enrichment in mitochondria, neuronal cell bodies, and synaptic components (**Figure 5B**). In the realm of molecular function, the target genes were significantly enriched in molecular function regulation, protein kinase activation, and oxidoreductase activity (**Figure 5C**). Further details of GO functional analysis results are shown in **Supplementary Table S6**. These target genes also demonstrated enrichment in KEGG pathways such as the ErbB signaling pathway, GnRH signaling pathway, TNF signaling pathway, and Fc epsilon RI signaling pathway (**Figure 5D**). Additional details of the KEGG pathway analysis results are provided in **Supplementary Table S7**. Intriguingly, cellular components such as neuron parts and mitochondrial components emerged as significantly related to this disease. These enriched pathways and terms significantly enhance our understanding of the role played by OS-DEGs in the onset and progression of BD.

### PPI network construction and hub genes identification

**Figure 6:**
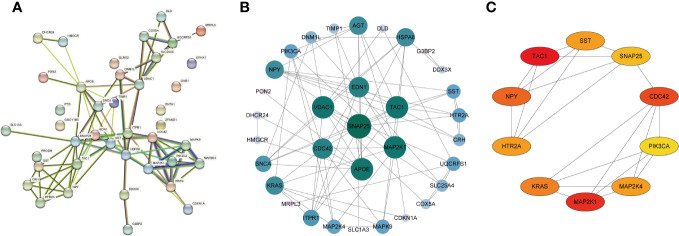
Construction of PPI network of the OS-DEGs. **(A)** PPI network was established by the OS-DEGs using STRING database. **(B)** The key subnetwork constructed by a second filtration via CytoNCA. **(C)** The PPI network’s top 10 hub genes are shown, ranked from red (high degree value) to yellow (low degree value), in order.

**Figure 7:**
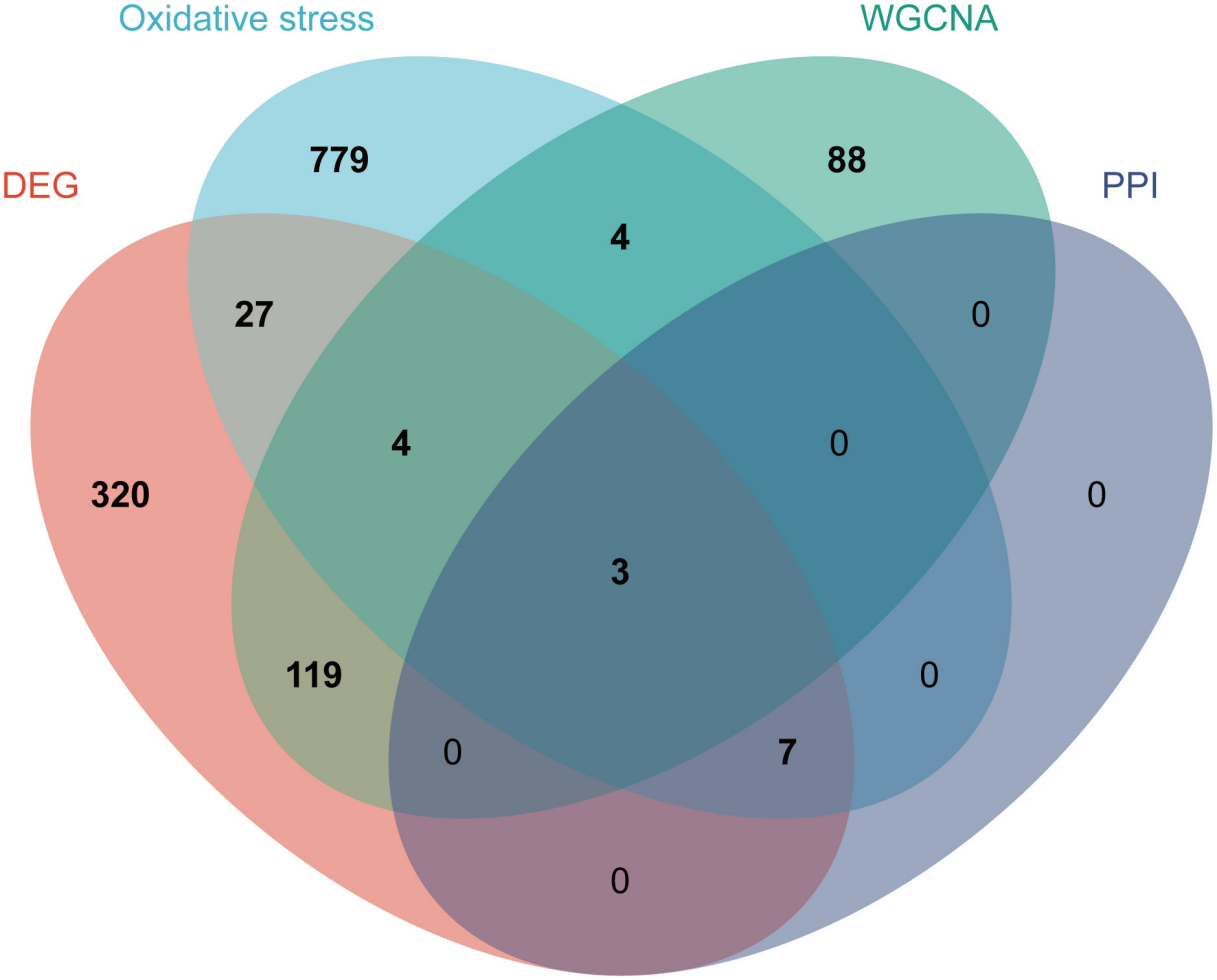
Identification of the hub genes. As shown by the venn diagram, the OS-DEGs, sienna 3 module genes of the WGCNA and the top ten genes in the PPI network were intersected in order to screen the three hub genes.

Subsequently, we uploaded the 41 OS-DEGs to the STRING database and constructed a PPI network to investigate their functionality. As illustrated in **Figure 6A**, the PPI network comprised 41 nodes (genes) and 82 edges (interactions), with a PPI enrichment *P-value* of 2.98e-11. A key subnetwork was established through a secondary filtration process via CytoNCA. Ultimately, 33 target-target interactions were identified, with SNAP25 identified as the central node within the entire network (**Figure 6B**). Meanwhile, the top ten genes from the PPI network were selected as potential hub genes based on Cytohubba analysis (**Figure 6C**). By extracting the intersection genes from the 480 DEGs, 218 sienna3 module genes, 824 OS-related genes, and the top 10 genes from the PPI network, we successfully identified three hub genes associated with OS in BD (**Figure 7**). **Table 3** provides detailed information about these hub genes.

**Table 3:** Details of the three hub genes.

Gene Abbreviation	Gene Name	adj. *P value*	Log FC
TAC1	tachykinin precursor 1	3.61119978594928e-07	-0.802113216699785
MAP2K1	mitogen-activated protein kinase kinase 1	0.001023431	-0.36591954
MAP2K4	mitogen-activated protein kinase kinase 4	0.000139922	-0.424934944

### Enrichment analysis of GSEA

**Figure 8:**
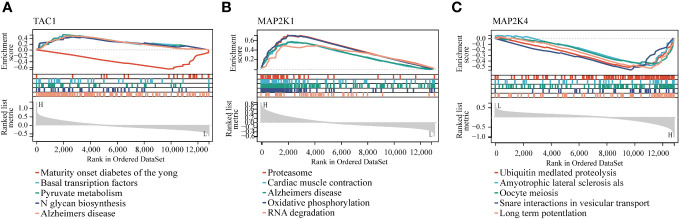
GSEA identified the three hub genes’ enriched pathways. **(A)** TAC1 **(B)** MAP2K1 **(C)** MAP2K4.

The GSEA results of these three hub genes showed that they are associated with a number of cellular activities which contain N glycan biosynthesis, RNA degradation and ubiquitin mediated proteolysis. In addition, GSEA of these genes showed that they are associated with several diseases, namely, Alzheimer’s disease, Amyotrophic lateral sclerosis als and maturity onset diabetes of the young (**Figure 8**).

### Expression validation of hub genes by RT-qPCR

**Figure 9:**
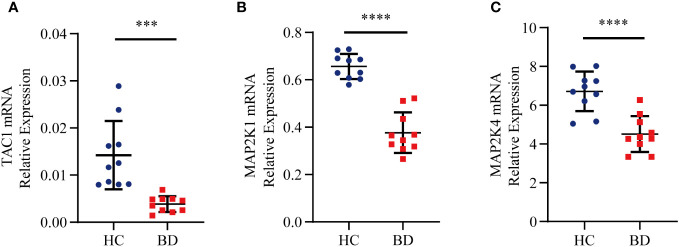
Relative expression levels of the three hub genes by RT-qPCR analysis. **(A)** TAC1. **(B)** MAP2K1. **(C)** MAP2K4. Significant differences were supposed at ***p-value < 0.001 **** *p-value* < 0.0001, compared with the control.

To more fully assess the levels of TAC1, MAP2K1 and MAP2K4in BD, we performed RT-qPCR to analyze mRNA expression of hub genes in the PBMCs from healthy individuals and BD patients (**Figure 9**). TAC1, MAP2K1 and MAP2K4 expression were found to be significantly down-regulated in BD compared to the normal cells (*P-value* < 0.001). The expression pattern matched the GSE5392 dataset.

### Validation of a diagnostic model

**Figure 10:**
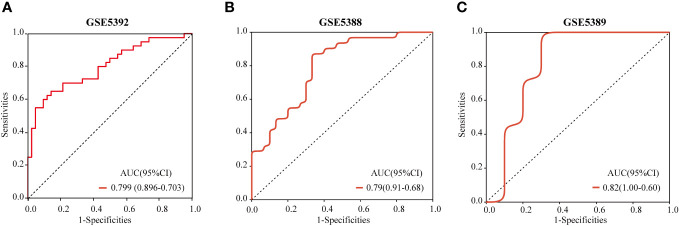
ROC curves and corresponding AUC values for each of the three expression cohorts. The post-mortem brain tissue samples from GSE5392 **(A)**, GSE5388 **(B)** and GSE5389 **(C)**.

The ROC curve was employed to validate the diagnostic model. The results demonstrated that the hub genes-based predictive model had effective diagnostic performance, with an AUC of 0.799 (**Figure 10A**). With AUCs 0.79 and 0.82, respectively, the models in GSE5388 and GSE5389 had reasonably high AUCs (**Figures 10B, C**).

## Discussion

Mounting evidence indicates that OS and disruptions in the antioxidant defense system play pivotal roles in the development and progression of BD (25). Brain cells are susceptible to oxidative stress and they lack antioxidant defenses to avoid excessive oxidative damage. Mitochondria, intracellular organelles responsible for ATP generation through oxidative phosphorylation in the electron transport chain, as well as for the synthesis of vital molecules and the orchestration of cellular reactions crucial to various cellular pathways and overall cellular functions, have emerged as central players in this context (26). Moreover, compelling evidence suggests that mitochondrial dysfunction and the disruption of quality control mechanisms substantially contribute to the accumulation of oxidized components within cells, as manifested in the brains of BD patients (27). Mitochondria, being both primary generators and immediate targets of ROS, trigger the activation of other OS pathways, such as neuroinflammation and disturbances in autophagy (27). Under stress conditions, excessive ROS production can produce excess mitochondrial fragments, impaired bioenergetics, and endogenous respiratory dysfunction leading to apoptosis (28). Researchers have discerned an enduring pathogenic cycle characterized by heightened OS, diminished electron transport chain (ETC) activity, dysregulation of the tricarboxylic acid (TCA) cycle, and reduced mitochondrial volumes in neurons derived from pluripotent stem cells (iPSCs) sourced from individuals with BD (29, 30). Indeed, mitochondrial dysfunction, primarily involving ETC disruption, is widely regarded as a principal driver of chronic OS in BD (31). The accrual of oxidative damage is presumed to contribute to neuronal cell demise through mechanisms involving apoptosis or the aggregation of oxidized proteins, thus impairing mood stabilization mechanisms (25). Consequently, it is plausible that mitochondrial dysfunction, as manifested by elevated OS, underlies the pathophysiology of BD (32). As our results demonstrate, cellular components such as neuronal cells and mitochondrial fractions exhibit significant associations with the disease. In a study of genomic expression in the cerebral cortex of patients with autism, schizophrenia, and BD, the clinical manifestations of individuals with different neuropsychiatric disorders were evaluated, and common genetic influences and common biological mechanisms were found to exist for these disorders, in which oxidative stress can influence neuronal apoptosis as a mediator of neuropsychiatric disorders (33). Another study showed that oxidative damage to lipids and proteins is associated with BD, with a significant increase in 4-hydroxyenal (4-HNE) protein adducts in the post-mortem anterior cingulate cortex of patients with BD. These adducts are the main products of lipid peroxidation, and when they accumulate in excess they are capable of inducing cytotoxic effects and disrupting cellular function (34).

To delve deeper into the profound effect of OS on the pathogenesis of BD, this study initially scrutinized RNA expression data from both BD patients and HCs to screen for OS-DEGs. GO enrichment analysis revealed that OS-DEGs predominantly exert their influence on mitochondria and synapses. ClueGO analysis further elucidated that OS-DEGs are intricately involved in processes such as the aerobic electron transport chain, regulating long-term neuronal synaptic plasticity, and the modulation of neuronal synaptic plasticity. These findings align with the established role of OS in the pathogenesis of BD (1). The brain, owing to its high oxygen utilization, relatively weak antioxidant defenses, and lipid-rich composition, is particularly vulnerable to oxidative damage (35). Neurons, abundant in mitochondria, are susceptible to disruptions in the ETC, culminating in metabolic disorders, oxidative stress, cellular damage, and inflammation (36). These factors collectively contribute to diminished neuronal repair and the translocation of mitochondria through the cytoskeleton to synaptic regions, further exacerbating neuronal dysfunction (31). Additionally, mitochondria play a pivotal in synaptic plasticity, and abnormal synaptic plasticity perpetuates mitochondrial dysfunction and reduced metabolism, thereby becoming an integral part of the pathogenesis of BD (37, 38). Concurrently, mitochondrial dysfunction triggers an ATP imbalance, affecting the Na^+^/K^+^ pump, which in turn impacts the action potential thresholds, leading to sustained neurotransmitter release and the manifestation of manic phases (39, 40).

Our findings suggest that the epidermal growth factor receptor (ErbB) signaling pathway and the gonadotropin-releasing hormone (GnRH) signaling pathway are the most critical KEGG pathways associated with OS-DEGs. The ErbB signaling pathway promotes the formation and maturation of excitatory synapses on GABAergic interneurons (41). Additionally, the disruption of myelin and dopaminergic function resulting from the loss of ErbB signaling in oligodendrocytes is the underlying mechanism of neuropsychiatric disorders (42). Investigation of transgenic mice in which ErbB signaling is blocked in oligodendrocytes *in vivo* results in changes to the number and morphology of oligodendrocytes decreased myelin thickness, and dopaminergic and behavioral alterations (43). Furthermore, certain studies indicate that mood disorders significantly enrich the GnRH signaling pathway, with a tendency toward strong upregulation (44). Given the intricate and multifaceted nature of the genetic pathways underlying BD, as discussed in the introduction, we utilized WGCNA to explore potential alterations in gene connectivity in BD (45).

Increasing evidences from gene expression studies suggested that the pathogenesis of BD were associated with altered expression of certain hub genes, and the measurement and analysis of gene expression information in post-mortem brain tissue from patients as well as clinical studies suggested that OS was the relevant molecular mechanism (46). In this study, we identified three hub genes (TAC1, MAP2K1, and MAP2K4) by intersecting DEGs, WGCNA, OS-related genes, and the key genes from the PPI network. These three hub genes exhibited strong diagnostic potential in the training dataset GSE5392 as well as external validation datasets GSE5388 and GSE5389. Notably, the mRNA expression levels of these three genes in the PBMCs of BD patients were markedly different. The TAC1 gene encodes sensory nerve neuropeptides substance P and neurokinin A (NKA), which play roles in neurogenesis, neuronutrition, neurotransmission, signal transduction, circadian rhythms, synaptic and myelin correlation, pathways and pathways and mechanisms that may have significance in pathophysiology (47). It is known that TAC1’s alpha, beta, and gamma splice variants all produce tachykinin neuropeptide substance P (48). Substance P-containing neurons and their receptors, which are abundantly expressed in the limbic system, including the amygdala, are involved in the regulation of mood (49). Evidence suggests that substance p could be expressed in the amygdala to control anxiety levels and pathological and physiological processes of mental illness (48, 50). Stress stimulation has been shown to elevate substance P levels and TAC1 mRNA in specific brain regions (51). Mice with TAC1 gene knockouts displayed reduced depressive and anxious behaviors as well as diminished nociception across a range of behavior tasks (52). Notably, their increased activity in depression-related paradigms, such as the forced-swimming test and the tail suspension test, as well as their lack of hyperactivity after bullectomy, can primarily be attributed to the TAC1 gene’s specific binding to substance P (53). Our multiple analyses indicate that neuropeptide genes play an important role in disorders associated with psychosis. In our study, MAP2K1 and MAP2K4 were identified as two additional hub genes involved in OS in the pathogenesis of BD. These genes encode proteins belonging to the dual specificity protein kinase family, which are essential components of the mitogen-activated protein kinases (MAPKs) signal transduction pathway (54). Research suggests that the mechanisms underlying increased peripheral inflammatory markers in BD may be linked to the differential activation of MAPKs (55). Positioned upstream of MAP kinases, MAP2K1 and MAP2K4 can activate MAP kinases in response to various extracellular and intracellular cues associated with synaptic transmission (56). The c-Jun NH2-terminal kinase (JNK) pathway is believed to be crucial for controlling neuronal survival and apoptosis in response to cerebral ischemia (57). MAP2K4 governs tumor development, apoptosis, the immune system, and inflammatory responses as a direct upstream activator of this pathway (58). MAP2K4 knockdown reduces JNK pathway activation and the production of inflammatory factors IL-6 and TNF-α (59). As revealed in our results, both cell-cell signaling and apoptotic processes are implicated in the pathogenesis of BD.

This study demonstrates that combining transcriptomics with clinical information can help improve future research and potential applications for clinical diagnosis. Inevitably, our study has some limitations. First, gene expression profiles may be affected by confounding factors, such as individual variability between samples, small sample sizes, and platforms. Therefore, more microarray data and stricter quality control need to be added to minimize errors. Second, a portion of the data used in our analysis was sourced from online databases. We obtained peripheral blood samples from only 10 BD patients and 10 HCs in the clinic due to the difficulty of obtaining autopsy brain tissue samples and the small clinical sample size of this single-center study. Our experiments provide preliminary evidence that OS processes are involved in the pathogenesis of BD, however the molecular mechanism of how it plays its role remains unclear. To validate the results of our analysis, we need to expand the sample size and conduct multi-center, large-sample randomized controlled studies and biological investigations at a later stage. Second, we did not investigate in depth the potential mechanisms through which these three hub genes function in BD. Future studies should explore the specific interactions between the hub genes and BD. In conclusion, the three hub genes we identified are directly or indirectly associated with OS in BD. Based on the limited preliminary data, our findings reveal an important role for OS in BD. However, the molecular mechanisms by which these three OS-related hub genes are involved in the progression of BD pathogenesis remain elusive and necessitate further investigation.

## Conclusion

Through a bioinformatics approach, we obtained three hub genes linking oxidative stress and BD. Exploring the biological processes and pathways they are involved in will help to comprehend the development of BD. Based on logistic regression analysis, we constructed a diagnostic model that can diagnose BD patients by detecting the expression of multiple genes in the blood. Although our findings support the conclusion that OS plays an important role in BD, there seemed to be some overlap with other psychiatric disorders, such as depression and schizophrenia (60), based on other work. However, our study incorporates further validation with blood samples from clinical patients, providing strong evidences to explore the involvement of oxidative stress in the pathogenesis of BD pathophysiology, which would be a new starting point.

## Data availability statement

Publicly available datasets were analyzed in this study. This data can be found here: GSE5392: https://www.ncbi.nlm.nih.gov/geo/query/acc.cgi?acc=GSE5392. GSE5388: https://www.ncbi.nlm.nih.gov/geo/query/acc.cgi?acc=GSE5388. GSE5389: https://www.ncbi.nlm.nih.gov/geo/query/acc.cgi?acc=GSE5389.

## Ethics statement

The studies involving humans were approved by the medical ethics committee of Shanxi Bethune Hospital. The studies were conducted in accordance with the local legislation and institutional requirements. The participants provided their written informed consent to participate in this study. The animal study was approved by the medical ethics committee of Shanxi Bethune Hospital. The study was conducted in accordance with the local legislation and institutional requirements. Written informed consent was obtained from the individual(s) for the publication of any potentially identifiable images or data included in this article.

## Author contributions

SW: Data curation, Investigation, Methodology, Software, Validation, Visualization, Writing – original draft, Writing – review & editing. YR: Conceptualization, Data curation, Formal analysis, Funding acquisition, Methodology, Visualization, Resources, Project administration, Writing – original draft. HH: Data curation, Investigation, Writing – review & editing. YL: Investigation, Writing – review & editing.
