# Supplementary material for: Exploring hub genes and crucial pathways linked to oxidative stress in bipolar disorder depressive episodes through bioinformatics analysis
Source: Front Psychiatry. 2024 Mar 6;15:1323527. doi: 10.3389/fpsyt.2024.1323527 (PMC10950934; doi:10.3389/fpsyt.2024.1323527)
Supplement: Supplementary file 1 [file Table_1.docx]

**Table S1: 824 genes related to Oxidative stress obtained from the Genecard website.**

| Gene Symbol | Relevance score |
| --- | --- |
| NOS3 | 64.56761932 |
| NOS2 | 58.54585648 |
| NOS1 | 46.5723114 |
| SOD1 | 46.12234497 |
| CAT | 45.53785706 |
| CPT2 | 42.39384842 |
| AIFM1 | 41.4300499 |
| CARS2 | 41.21313095 |
| TNF | 39.15766144 |
| ELAC2 | 38.51987457 |
| FARS2 | 38.5039444 |
| NFE2L2 | 37.95635986 |
| TP53 | 37.58535767 |
| HMOX1 | 37.24214172 |
| OXSR1 | 35.07815933 |
| GFM1 | 34.88779068 |
| AARS2 | 34.43948364 |
| SOD2 | 34.02082062 |
| MAPK14 | 32.24443436 |
| GSR | 32.00449753 |
| OSGIN1 | 31.84185028 |
| MAPK8 | 31.05522919 |
| XDH | 30.14569473 |
| OSER1 | 29.73036385 |
| MPO | 29.36198807 |
| IL6 | 28.28831482 |
| TXN | 28.19101715 |
| TUFM | 28.03699875 |
| OSGIN2 | 27.35177612 |
| POLR1C | 27.33596802 |
| MAPK1 | 26.78823471 |
| TSFM | 26.46313095 |
| PARK7 | 26.13332748 |
| OLR1 | 25.91897964 |
| MTRFR | 25.9104805 |
| IL1B | 25.44779968 |
| G6PD | 25.11792946 |
| TXN2 | 24.99613571 |
| VARS2 | 24.88299751 |
| PNPT1 | 24.87753868 |
| SIRT1 | 24.37094879 |
| CYCS | 24.02589607 |
| CASP3 | 23.97985077 |
| RYR2 | 23.82392693 |
| GPX1 | 23.50757599 |
| NQO1 | 23.12179565 |
| APP | 23.02246857 |
| CRP | 23.00228691 |
| MTFMT | 22.67368507 |
| PTGS2 | 22.45686722 |
| PON1 | 22.43655777 |
| CXCL8 | 22.13799286 |
| HSPA5 | 22.12746811 |
| GTPBP3 | 22.11974907 |
| ACADVL | 21.93836021 |
| MAP3K5 | 21.90445518 |
| ADPRS | 21.82743835 |
| SLC6A4 | 21.70595741 |
| NOS1AP | 21.51211929 |
| CRH | 21.39337158 |
| HADHA | 21.34871483 |
| MRPL44 | 21.33223534 |
| CYBA | 21.32664108 |
| CALM1 | 21.12927055 |
| FOXO3 | 21.12760353 |
| JUN | 21.10403442 |
| GSTM1 | 21.07699585 |
| CCL2 | 21.07436371 |
| INS | 21.02113342 |
| HADHB | 20.94484329 |
| G3BP1 | 20.84153748 |
| OGG1 | 20.72131157 |
| XBP1 | 20.68072319 |
| DDIT3 | 20.52202988 |
| GFM2 | 20.33967972 |
| FOXO1 | 20.26579285 |
| TARS2 | 20.18214417 |
| ATF4 | 20.18185043 |
| RYR1 | 20.08107185 |
| PRDX2 | 20.02110672 |
| GSTP1 | 19.99526596 |
| ALB | 19.94689178 |
| KEAP1 | 19.82452393 |
| AKT1 | 19.65703773 |
| HSF1 | 19.64547729 |
| PARP1 | 19.54890442 |
| MSRA | 19.42743683 |
| EARS2 | 19.42544365 |
| CYP2D6 | 19.40511894 |
| EIF2AK3 | 19.34880066 |
| EDN1 | 19.29745102 |
| CYBB | 19.21492767 |
| HSP90AA1 | 19.20866776 |
| FASTKD2 | 19.19743156 |
| IFNG | 19.1658535 |
| HSPB1 | 19.11595154 |
| PRKN | 19.0843811 |
| CYP3A4 | 19.0650444 |
| TRDN | 19.0202713 |
| GAPDH | 18.99566269 |
| ACADM | 18.99215698 |
| BCL2 | 18.97240829 |
| PRDX5 | 18.89130592 |
| APOE | 18.85571098 |
| MAPK10 | 18.80795288 |
| MRPS34 | 18.79366302 |
| CPT1A | 18.77950287 |
| ESR1 | 18.66530228 |
| CASQ2 | 18.55911255 |
| EIF2S1 | 18.53469658 |
| HSPA4 | 18.50715637 |
| BDNF | 18.48884964 |
| ATP5F1A | 18.3808403 |
| VWF | 18.34720039 |
| IL10 | 18.23929024 |
| FMO3 | 18.21386147 |
| TGFB1 | 18.06455231 |
| APEX1 | 18.04248428 |
| HSPA1A | 18.0305748 |
| STIP1 | 17.9945488 |
| CYP1A2 | 17.94526863 |
| QRSL1 | 17.83962631 |
| MTO1 | 17.82758331 |
| ADIPOQ | 17.81201935 |
| VEGFA | 17.7965126 |
| HIF1A | 17.78186035 |
| C1QBP | 17.76380348 |
| SNCA | 17.74045753 |
| ACE | 17.5567627 |
| MAOA | 17.32284164 |
| NFKB1 | 17.11958504 |
| NR3C1 | 17.07144928 |
| HSD17B4 | 17.06520844 |
| ABL1 | 17.03106499 |
| CAV1 | 16.87313271 |
| CYP1A1 | 16.85873413 |
| PRDX6 | 16.81410217 |
| BAX | 16.80002022 |
| GSTT1 | 16.70978165 |
| HBG2 | 16.65698242 |
| SELENON | 16.61539459 |
| NFS1 | 16.59470749 |
| MAPK9 | 16.56547928 |
| PLA2G7 | 16.37930679 |
| G3BP2 | 16.299757 |
| ICAM1 | 16.29804039 |
| SOD3 | 16.29636955 |
| CP | 16.28851891 |
| MRPS16 | 16.18807793 |
| SLC25A20 | 16.18043137 |
| SERP1 | 16.1289959 |
| MRPL12 | 16.0827446 |
| ACADS | 16.04718399 |
| PPARG | 15.92518997 |
| NUDT1 | 15.90061569 |
| CYP2E1 | 15.86891937 |
| CALM3 | 15.81505394 |
| PRKCD | 15.78303051 |
| SHC1 | 15.7647934 |
| TLR4 | 15.68749046 |
| ACOX1 | 15.6396637 |
| PTCD3 | 15.58426666 |
| PRDX3 | 15.49214554 |
| SQSTM1 | 15.46032906 |
| EHHADH | 15.44819069 |
| NDUFS4 | 15.41652393 |
| MAOB | 15.4156332 |
| ATF6 | 15.4099884 |
| ATM | 15.33698177 |
| SDHA | 15.30450249 |
| VCAM1 | 15.17819595 |
| CAV3 | 15.07686615 |
| PRDX1 | 15.02237225 |
| GATB | 15.00973225 |
| LONP1 | 14.97823334 |
| TRPM2 | 14.97472191 |
| KNG1 | 14.96832561 |
| OXR1 | 14.87685776 |
| MAPK3 | 14.83343887 |
| POMC | 14.69797421 |
| COX5A | 14.62393951 |
| PINK1 | 14.59579277 |
| UCP2 | 14.50431252 |
| ACADL | 14.50081539 |
| MTOR | 14.46589756 |
| LYRM4 | 14.4475832 |
| CLU | 14.43998337 |
| PPARGC1A | 14.42654419 |
| HADH | 14.37486649 |
| ERN1 | 14.35173321 |
| GPX3 | 14.26064968 |
| HSPA8 | 14.25354004 |
| FOS | 14.21327209 |
| ACAD9 | 14.1696043 |
| JAK2 | 14.13451958 |
| GATC | 14.12934113 |
| ALDH2 | 14.09181881 |
| MIPEP | 14.0760498 |
| NOX4 | 14.06315804 |
| MICOS13 | 14.05753231 |
| POLRMT | 14.05377388 |
| GPX7 | 14.05315018 |
| VARS1 | 14.04933739 |
| APOA1 | 14.03872776 |
| KCNJ5 | 14.00282192 |
| PDE5A | 13.99831581 |
| HSD17B10 | 13.97479916 |
| ETFDH | 13.97140884 |
| PRKAA1 | 13.96848106 |
| LEP | 13.96389389 |
| PRKAA2 | 13.92602158 |
| CRHR1 | 13.8766489 |
| CYP2C9 | 13.84357834 |
| SDHB | 13.77776527 |
| EIF2AK2 | 13.76196766 |
| MSRB2 | 13.75652313 |
| ADRB2 | 13.75154495 |
| EIF2AK1 | 13.74783707 |
| NPY | 13.68275356 |
| SNTA1 | 13.66604614 |
| SDHD | 13.56152344 |
| ABCD1 | 13.55340099 |
| GCH1 | 13.5491848 |
| CD36 | 13.53317833 |
| AGT | 13.52801323 |
| TXNIP | 13.51115513 |
| NDUFS8 | 13.48498344 |
| MAP2K4 | 13.46408081 |
| PTGS1 | 13.41687298 |
| CACNA1C | 13.39611435 |
| MB | 13.38922596 |
| CYP2C19 | 13.33522797 |
| COMT | 13.30656624 |
| MRPS7 | 13.29753399 |
| CASP9 | 13.29557991 |
| TRMT10C | 13.27902317 |
| TIA1 | 13.26512527 |
| SCN5A | 13.2596674 |
| CDKN1A | 13.24277687 |
| BLVRB | 13.21268654 |
| CYP1B1 | 13.20862007 |
| P4HB | 13.191082 |
| HBB | 13.13804054 |
| MRPS22 | 13.11743736 |
| PPARA | 13.06686592 |
| NOX1 | 13.06271362 |
| GLRX | 13.05428982 |
| FMO1 | 13.04918957 |
| STAT3 | 13.02546692 |
| MRPL3 | 12.99912739 |
| SELE | 12.97361946 |
| SP1 | 12.93963337 |
| EME2 | 12.91359901 |
| NCF2 | 12.91092682 |
| MSRB1 | 12.87435913 |
| HMGB1 | 12.81447887 |
| C2orf69 | 12.80226135 |
| OXT | 12.78759575 |
| AGTR1 | 12.78602219 |
| MAP2K6 | 12.76526546 |
| SLC2A1 | 12.75129509 |
| PRDX4 | 12.71737671 |
| ALOX5 | 12.71004105 |
| HSPA9 | 12.68583679 |
| TXNRD1 | 12.64864922 |
| CALM2 | 12.62694263 |
| PON2 | 12.62674713 |
| KCNH2 | 12.6235466 |
| MDM2 | 12.60835266 |
| MAPKAPK2 | 12.59175777 |
| MAPK11 | 12.52666855 |
| EPO | 12.5091877 |
| ATF2 | 12.50110054 |
| ETFA | 12.46753216 |
| MRPS25 | 12.45834064 |
| MMP2 | 12.4520998 |
| IL1A | 12.40802574 |
| CHAT | 12.36677361 |
| POR | 12.32226849 |
| SLC25A4 | 12.31113434 |
| CREB1 | 12.30975533 |
| TPO | 12.30744934 |
| HMOX2 | 12.29500771 |
| STK25 | 12.29452324 |
| VCP | 12.25754356 |
| SRC | 12.24508667 |
| SERPINE1 | 12.23010254 |
| UCP3 | 12.22106934 |
| AGER | 12.16032219 |
| SELP | 12.15613651 |
| RHOA | 12.13817215 |
| MRPS2 | 12.10433102 |
| KCNQ1 | 12.10216522 |
| CYGB | 12.0895052 |
| VHL | 12.08387375 |
| FKBP5 | 12.0670023 |
| MRPS28 | 11.98732567 |
| OXTR | 11.98439789 |
| PSEN1 | 11.94860268 |
| MRPS23 | 11.85874748 |
| SGCB | 11.83583546 |
| CBS | 11.78616238 |
| MAP2K3 | 11.78332043 |
| TIMM22 | 11.752244 |
| DDAH2 | 11.73122978 |
| ADCYAP1 | 11.7115593 |
| GPX4 | 11.71119118 |
| NOSTRIN | 11.70561314 |
| EPHX1 | 11.70028019 |
| PRKG1 | 11.67440414 |
| SESN2 | 11.67201805 |
| PIK3CG | 11.65028191 |
| FAS | 11.64316654 |
| ETFB | 11.6289053 |
| HERPUD1 | 11.6059761 |
| ARG1 | 11.5912056 |
| BRCA1 | 11.58319664 |
| NPPA | 11.58304405 |
| MRPS14 | 11.57415199 |
| GCLC | 11.56419563 |
| IGF1 | 11.55511189 |
| KCNE1 | 11.55125904 |
| CS | 11.55007744 |
| TYR | 11.53114128 |
| ATP2A2 | 11.47511482 |
| NFE2L1 | 11.45898247 |
| GGT1 | 11.45829391 |
| TF | 11.4497757 |
| HTRA2 | 11.43383026 |
| ADH5 | 11.36740875 |
| TXNRD2 | 11.35335922 |
| IL1RN | 11.31184196 |
| ATF3 | 11.30884743 |
| UQCRFS1 | 11.30656242 |
| IL18 | 11.30206299 |
| PRORP | 11.28495121 |
| MAPT | 11.27036381 |
| KDR | 11.21539116 |
| SIRT3 | 11.2124939 |
| LMNA | 11.19380569 |
| NOSIP | 11.19130707 |
| PRKCB | 11.18237209 |
| MT-CYB | 11.10756111 |
| MMP9 | 11.10374641 |
| RPS6KA5 | 11.0865612 |
| GFER | 11.08452606 |
| SRXN1 | 11.07930374 |
| ANXA5 | 11.03054047 |
| ARG2 | 11.01819324 |
| TERT | 11.00774574 |
| TARDBP | 10.99129581 |
| LOC110806262 | 10.96341896 |
| ANK2 | 10.94432545 |
| MAP2K1 | 10.91274452 |
| MAP2K7 | 10.85728073 |
| REN | 10.84833145 |
| BCL2L1 | 10.84772778 |
| NGB | 10.83638763 |
| ASL | 10.82748222 |
| GSTA1 | 10.81641579 |
| NR3C2 | 10.81021309 |
| BRCA2 | 10.80265999 |
| PDHA1 | 10.79341698 |
| VIP | 10.77510738 |
| EGFR | 10.77001381 |
| PCNA | 10.76415062 |
| HP | 10.74984455 |
| GHRL | 10.74633598 |
| GLRX2 | 10.74102783 |
| IL13 | 10.73296738 |
| MT-CO1 | 10.72823524 |
| EP300 | 10.72307205 |
| KRIT1 | 10.72057152 |
| NDUFS1 | 10.71494102 |
| PRKD1 | 10.70860004 |
| PIK3CA | 10.68001938 |
| NDUFS2 | 10.67818642 |
| HTR2A | 10.64382458 |
| HYOU1 | 10.62556648 |
| DYNLL1 | 10.61842346 |
| MTHFR | 10.61399078 |
| PTEN | 10.58253288 |
| FXN | 10.57295799 |
| RAC1 | 10.55184937 |
| STAT1 | 10.5514183 |
| NPPB | 10.54782104 |
| CRYAB | 10.51087952 |
| CDKN2A | 10.5069313 |
| MAPK13 | 10.49020576 |
| DBH | 10.47676373 |
| PPP1R15A | 10.47542 |
| SUOX | 10.45798206 |
| SIRT6 | 10.45716095 |
| GCLM | 10.4403801 |
| CALR | 10.43935776 |
| CYP2B6 | 10.43680573 |
| DRD2 | 10.41300964 |
| GSS | 10.39466953 |
| FMO2 | 10.38799095 |
| HSPD1 | 10.36175156 |
| NCF1 | 10.34266376 |
| AKR1A1 | 10.33870792 |
| NFKBIA | 10.33488369 |
| CALCA | 10.32987404 |
| H6PD | 10.32581711 |
| SIRT2 | 10.32369709 |
| GLUD1 | 10.22729778 |
| PRL | 10.22009087 |
| NLRP3 | 10.21752739 |
| SFXN4 | 10.21462059 |
| HSP90B1 | 10.19292641 |
| GJA1 | 10.19153881 |
| DUSP1 | 10.17041683 |
| CUL3 | 10.15491581 |
| PTK2B | 10.14657497 |
| NDUFV1 | 10.14627075 |
| TLR2 | 10.14410973 |
| MUTYH | 10.08191395 |
| CACNA1S | 10.07115555 |
| MGST1 | 10.06913757 |
| CEBPB | 10.04976654 |
| TTN | 10.00460243 |
| BMP6 | 9.978807449 |
| NTHL1 | 9.956516266 |
| RORA | 9.946562767 |
| MT-ND1 | 9.934121132 |
| CNR1 | 9.923035622 |
| PRODH | 9.920479774 |
| TH | 9.900054932 |
| ASS1 | 9.891366005 |
| THBD | 9.8552351 |
| POLG | 9.828689575 |
| EDNRA | 9.826086998 |
| SDHC | 9.815465927 |
| PGD | 9.80252552 |
| CYP11B2 | 9.788980484 |
| CFTR | 9.786216736 |
| MAPKAPK3 | 9.782697678 |
| SCP2 | 9.775310516 |
| ALDH3A2 | 9.77331543 |
| PRKCA | 9.766319275 |
| UCP1 | 9.757102966 |
| SCARA3 | 9.747265816 |
| MIEF2 | 9.737203598 |
| FDXR | 9.734016418 |
| OGDH | 9.734002113 |
| EGF | 9.730560303 |
| BCL2L11 | 9.726078033 |
| CRAT | 9.72063446 |
| CHUK | 9.719555855 |
| SMPD1 | 9.701366425 |
| PRKAB1 | 9.677200317 |
| MYH7 | 9.67407608 |
| DDAH1 | 9.669694901 |
| CASP8 | 9.64680481 |
| ADM | 9.640275955 |
| CYC1 | 9.63439846 |
| HSPA1B | 9.622514725 |
| NOTCH3 | 9.620920181 |
| AKR1B1 | 9.617060661 |
| HFE | 9.603301048 |
| ERCC8 | 9.60195446 |
| NDUFB9 | 9.571755409 |
| FMO4 | 9.57080555 |
| NDUFA6 | 9.544947624 |
| EGR1 | 9.530410767 |
| EEF1A1 | 9.512599945 |
| ACP1 | 9.492101669 |
| ISCU | 9.486057281 |
| TNFRSF1A | 9.437876701 |
| THBS1 | 9.435383797 |
| PTPN1 | 9.423009872 |
| TSPO | 9.3954916 |
| S100A8 | 9.391409874 |
| BAK1 | 9.38435173 |
| DLD | 9.36259079 |
| GSK3B | 9.356373787 |
| LOX | 9.347177505 |
| STK4 | 9.342155457 |
| LPO | 9.337626457 |
| DHCR24 | 9.33490181 |
| SMAD3 | 9.291467667 |
| NDUFS6 | 9.291022301 |
| ACTA1 | 9.289494514 |
| GLO1 | 9.240427017 |
| NRF1 | 9.233442307 |
| BMP2 | 9.226184845 |
| ALDH1A1 | 9.203494072 |
| PPIA | 9.178285599 |
| APOB | 9.162621498 |
| CDK2 | 9.162601471 |
| NOL3 | 9.122794151 |
| DECR1 | 9.119390488 |
| MAPK12 | 9.100322723 |
| SETD2 | 9.076541901 |
| HRAS | 9.075181007 |
| LOC110973015 | 9.069489479 |
| CYP3A5 | 9.068586349 |
| FN1 | 9.066802025 |
| ATR | 9.061602592 |
| INSR | 9.059459686 |
| RPS27A | 9.058636665 |
| AOC3 | 9.046955109 |
| ATP1A3 | 9.032586098 |
| ECHS1 | 9.032548904 |
| NOD2 | 9.03037262 |
| IL2 | 9.027671814 |
| CDKN3 | 9.004052162 |
| NEIL1 | 8.991065979 |
| CTSB | 8.988645554 |
| CYP2C8 | 8.987356186 |
| AR | 8.967414856 |
| SGK1 | 8.964550018 |
| FAM120A | 8.96222496 |
| ZFAND1 | 8.957968712 |
| MT-CO3 | 8.931141853 |
| FKBP4 | 8.913124084 |
| GUCY1A1 | 8.910669327 |
| CYP2A6 | 8.898550034 |
| TIMP1 | 8.896763802 |
| MDH2 | 8.861497879 |
| GCDH | 8.852517128 |
| ALDH9A1 | 8.845870018 |
| PRNP | 8.83900547 |
| GSTM3 | 8.835626602 |
| DAXX | 8.834309578 |
| STUB1 | 8.833026886 |
| CYP4F2 | 8.829750061 |
| ELN | 8.829155922 |
| CTNNB1 | 8.827878952 |
| PAH | 8.797477722 |
| NDUFS3 | 8.766843796 |
| AKAP9 | 8.735495567 |
| NDUFV2 | 8.731063843 |
| SST | 8.714668274 |
| MSRB3 | 8.687809944 |
| AHSP | 8.681792259 |
| SYK | 8.681617737 |
| ERO1A | 8.681602478 |
| UGT1A1 | 8.679016113 |
| CYP27A1 | 8.678217888 |
| IDH1 | 8.674365044 |
| ALOX15 | 8.670619011 |
| KCNE2 | 8.668094635 |
| LOC111365141 | 8.662199974 |
| IKBKB | 8.657339096 |
| MICB | 8.649765015 |
| SLC22A5 | 8.646911621 |
| SESN1 | 8.646465302 |
| EPRS1 | 8.641983032 |
| HTT | 8.636824608 |
| PLA2G6 | 8.629741669 |
| PLG | 8.629447937 |
| CRYAA | 8.608531952 |
| TRPA1 | 8.604146957 |
| MTR | 8.590560913 |
| IL17A | 8.582487106 |
| TMEM161A | 8.571249008 |
| HSP90AB1 | 8.565980911 |
| ALAD | 8.565149307 |
| SCN4A | 8.563477516 |
| EIF4E | 8.56114006 |
| LRRK2 | 8.541936874 |
| PTK2 | 8.533121109 |
| TRPV1 | 8.528167725 |
| ALOX12 | 8.519989967 |
| ELAVL1 | 8.507261276 |
| PPARD | 8.501559258 |
| FAAH | 8.500638962 |
| NDUFS7 | 8.481273651 |
| TPH1 | 8.477363586 |
| ABCC1 | 8.471549034 |
| FANCD2 | 8.470567703 |
| PLCG2 | 8.465331078 |
| GPX2 | 8.453689575 |
| RAC2 | 8.440721512 |
| SERPINA1 | 8.438632011 |
| GUCY1B1 | 8.429702759 |
| CDC42 | 8.42941761 |
| NDUFB10 | 8.428587914 |
| ELANE | 8.422706604 |
| CCND1 | 8.422581673 |
| TRAF2 | 8.415679932 |
| HAO1 | 8.414172173 |
| MYC | 8.409730911 |
| CCL3 | 8.408350945 |
| CASP2 | 8.395397186 |
| HBA1 | 8.377786636 |
| MMP1 | 8.375926971 |
| CLEC4A | 8.36697197 |
| AKR1C1 | 8.361832619 |
| FASLG | 8.360376358 |
| MT-ATP6 | 8.356040955 |
| UQCRQ | 8.348356247 |
| C9orf72 | 8.345028877 |
| FMR1 | 8.338254929 |
| COX4I1 | 8.32827282 |
| ZFP36 | 8.323566437 |
| PLA2G4A | 8.319339752 |
| XRCC1 | 8.318602562 |
| CDK5 | 8.275880814 |
| VDR | 8.268777847 |
| TGFB2 | 8.26026535 |
| F5 | 8.257845879 |
| BCHE | 8.24929142 |
| IGF2BP1 | 8.234205246 |
| GADD45A | 8.232183456 |
| DSPP | 8.227081299 |
| CRHR2 | 8.226239204 |
| PIK3R1 | 8.216609955 |
| AOX1 | 8.216506004 |
| SMAD2 | 8.213378906 |
| DDX3X | 8.21159935 |
| DUOX2 | 8.211116791 |
| BAD | 8.2076931 |
| PECAM1 | 8.201183319 |
| ALDH3A1 | 8.201002121 |
| ACO1 | 8.197734833 |
| S100A9 | 8.192889214 |
| CYP19A1 | 8.188124657 |
| TG | 8.181111336 |
| PTS | 8.179668427 |
| MIF | 8.178840637 |
| GPT | 8.166996002 |
| MAPKAP1 | 8.160721779 |
| PRKCZ | 8.152323723 |
| MYLK | 8.150840759 |
| NDRG1 | 8.143901825 |
| YARS2 | 8.136387825 |
| CDKN1B | 8.105861664 |
| F2 | 8.090478897 |
| NOA1 | 8.07502079 |
| CASP1 | 8.069431305 |
| MMP3 | 8.068405151 |
| IL4 | 8.067857742 |
| PDIA2 | 8.067380905 |
| IRS1 | 8.057025909 |
| KLF2 | 8.056524277 |
| GBA | 8.050409317 |
| AS3MT | 8.047196388 |
| CCL11 | 8.039859772 |
| CREB3 | 8.027519226 |
| CYP17A1 | 8.006484985 |
| CHEK1 | 8.005970001 |
| RELA | 8.003908157 |
| GSTA4 | 7.998653412 |
| EPX | 7.993919849 |
| AGRN | 7.993590832 |
| CYP24A1 | 7.977929115 |
| CTSD | 7.964879036 |
| TACR1 | 7.952466965 |
| APOH | 7.948179245 |
| GLUL | 7.932922363 |
| SCN4B | 7.931811333 |
| RARA | 7.922787666 |
| SDHAF1 | 7.920912743 |
| GSTM2 | 7.91892004 |
| GPX8 | 7.917350292 |
| ALKBH1 | 7.916387558 |
| EIF2B5 | 7.906721115 |
| IKBKG | 7.905540943 |
| TNFRSF1B | 7.899206161 |
| NDUFC2 | 7.898485184 |
| LTF | 7.891988754 |
| TRAF6 | 7.881187916 |
| NGF | 7.878628731 |
| CPT1B | 7.87501049 |
| ADRB1 | 7.8652668 |
| ABCB1 | 7.85589695 |
| PLAT | 7.852129459 |
| MT-CO2 | 7.846092224 |
| DUSP19 | 7.824460983 |
| EIF2AK4 | 7.810894966 |
| ACOX2 | 7.80105257 |
| CYP11A1 | 7.794293404 |
| MT-ND3 | 7.782655239 |
| SCARB1 | 7.760556698 |
| IDO1 | 7.75843811 |
| EPHX2 | 7.756476402 |
| CREBBP | 7.746898174 |
| SLC18A3 | 7.745666981 |
| GLA | 7.740231514 |
| PPOX | 7.736665249 |
| ABCA1 | 7.733223915 |
| CYP2J2 | 7.703573227 |
| CASP4 | 7.700765133 |
| DMD | 7.700355053 |
| ITPR1 | 7.698207855 |
| TRPV4 | 7.690088272 |
| PXN | 7.683664322 |
| ADH7 | 7.6822896 |
| RPS6KA4 | 7.679182053 |
| PLA2G2A | 7.676521301 |
| E2F1 | 7.674899578 |
| MT-TL1 | 7.673847675 |
| IL1R1 | 7.671483517 |
| NDUFAF2 | 7.667541981 |
| CCN2 | 7.667165279 |
| GSTA2 | 7.659856796 |
| KCNMA1 | 7.65194416 |
| ERCC6 | 7.650299072 |
| PLAU | 7.644456863 |
| TAC1 | 7.638101578 |
| PC | 7.636672974 |
| PRKD2 | 7.632889748 |
| LIAS | 7.631311417 |
| UGT1A6 | 7.585700989 |
| PTPN11 | 7.582022667 |
| MIR21 | 7.573892593 |
| F8 | 7.572912216 |
| PRKCG | 7.564688683 |
| GSTO1 | 7.554427147 |
| TREM2 | 7.553792953 |
| MAPK8IP1 | 7.553456783 |
| PPIF | 7.551187038 |
| SERPINF1 | 7.542769432 |
| JUNB | 7.539649963 |
| ALKBH2 | 7.533986092 |
| HDAC2 | 7.529509068 |
| TP53INP1 | 7.527973175 |
| ASPH | 7.520893097 |
| ATP5MK | 7.511624813 |
| APAF1 | 7.506846428 |
| BCKDHB | 7.503805161 |
| SNAP25 | 7.502079964 |
| ACAA1 | 7.497674942 |
| NOTCH1 | 7.489213943 |
| MT-ND4 | 7.481687546 |
| RXRA | 7.479607582 |
| SLC17A5 | 7.46630621 |
| IGF1R | 7.462335587 |
| FOXO4 | 7.461912155 |
| NDUFA9 | 7.460895061 |
| MAP2K2 | 7.460360527 |
| SLC4A1 | 7.455060482 |
| DNM2 | 7.45341444 |
| AHR | 7.452066422 |
| MANF | 7.446428299 |
| GDF15 | 7.444869041 |
| NDUFA8 | 7.440505981 |
| CASP7 | 7.437493801 |
| RETN | 7.428315639 |
| CAMK2G | 7.426632881 |
| OPRM1 | 7.424616814 |
| BDKRB2 | 7.421019554 |
| PIEZO1 | 7.400797844 |
| RAF1 | 7.394124031 |
| MUC1 | 7.382254601 |
| EIF4G1 | 7.380327225 |
| DYRK3 | 7.371419907 |
| DNMT1 | 7.36651516 |
| SLC1A3 | 7.36584568 |
| TLR9 | 7.363637447 |
| ACHE | 7.35682106 |
| PTGIS | 7.348956585 |
| RPA1 | 7.343302727 |
| FLT1 | 7.333851814 |
| COX6B1 | 7.332556725 |
| ITGAM | 7.330077648 |
| SLC8A1 | 7.328814507 |
| KRAS | 7.314034462 |
| STK24 | 7.313504696 |
| CXCL1 | 7.30454874 |
| MAP3K7 | 7.297404289 |
| NR2C2 | 7.291337013 |
| NPM1 | 7.290407658 |
| SLC25A1 | 7.289483547 |
| CYP3A7 | 7.280613422 |
| COA8 | 7.272818565 |
| MSR1 | 7.267974854 |
| CHCHD2 | 7.263921738 |
| RB1 | 7.24966383 |
| CAPN3 | 7.247086048 |
| CCL5 | 7.244622231 |
| CSF1 | 7.237958908 |
| GUCY1A2 | 7.236926079 |
| FTO | 7.236349583 |
| NAMPT | 7.230423927 |
| TFRC | 7.226506233 |
| FGF2 | 7.222884178 |
| MAP3K1 | 7.222108841 |
| DSP | 7.218204975 |
| VIM | 7.217618942 |
| ODC1 | 7.215794086 |
| CHKA | 7.210188389 |
| AMBP | 7.2063694 |
| SLC6A3 | 7.201037407 |
| ITGB1 | 7.198799133 |
| NDUFA1 | 7.195775509 |
| ENDOG | 7.181473732 |
| DNM1L | 7.178201675 |
| GRB2 | 7.176875114 |
| CD40 | 7.17415905 |
| HMGCR | 7.16720295 |
| NDUFA10 | 7.166172981 |
| QDPR | 7.16564846 |
| ADCY5 | 7.161778927 |
| CPOX | 7.134279728 |
| AKR1C3 | 7.131139278 |
| MCL1 | 7.111439705 |
| XIAP | 7.106122017 |
| CYP4A11 | 7.100946903 |
| ATP5F1E | 7.10088253 |
| MBL2 | 7.098783493 |
| VDAC1 | 7.097242355 |
| DNAH8 | 7.097195625 |
| CYB5R3 | 7.089167595 |
| PTPN3 | 7.081127167 |
| MT-ND5 | 7.079634666 |
| S100B | 7.077723503 |
| FH | 7.076803207 |
| TNFSF10 | 7.068021297 |
| RCAN1 | 7.062831879 |
| HRH2 | 7.057354927 |
| BACE1 | 7.055316925 |
| PDIA3 | 7.05450058 |
| ALPP | 7.047825813 |
| PDLIM4 | 7.046460629 |
| PARG | 7.042935848 |
| RPTOR | 7.038090706 |
| NDUFAF1 | 7.037741184 |
| ACO2 | 7.033308983 |
| NDUFB8 | 7.031628132 |
| PLCG1 | 7.026249409 |
