# Supplementary material for: Exploring hub genes and crucial pathways linked to oxidative stress in bipolar disorder depressive episodes through bioinformatics analysis
Source: Front Psychiatry. 2024 Mar 6;15:1323527. doi: 10.3389/fpsyt.2024.1323527 (PMC10950934; doi:10.3389/fpsyt.2024.1323527)
Supplement: Supplementary file 6 [file Table_6.docx]

**Table S6 GO enrichment analysis.**

| Ontology | ID | Description | GeneRatio | *p-value* | *p-adjust* | *q-value* | Count |
| --- | --- | --- | --- | --- | --- | --- | --- |
| BP | GO:0007268 | chemical synaptic transmission | 15/41 | 1.44E-12 | 1.31E-09 | 6.05E-10 | 15 |
| BP | GO:0098916 | anterograde trans-synaptic signaling | 15/41 | 1.44E-12 | 1.31E-09 | 6.05E-10 | 15 |
| BP | GO:0099537 | trans-synaptic signaling | 15/41 | 1.63E-12 | 1.31E-09 | 6.05E-10 | 15 |
| BP | GO:0099536 | synaptic signaling | 15/41 | 1.83E-12 | 1.31E-09 | 6.05E-10 | 15 |
| BP | GO:0007267 | cell-cell signaling | 20/41 | 1.40E-11 | 7.99E-09 | 3.68E-09 | 20 |
| BP | GO:0008219 | cell death | 23/41 | 1.81E-11 | 8.64E-09 | 3.98E-09 | 23 |
| BP | GO:0008015 | blood circulation | 13/41 | 8.08E-11 | 3.14E-08 | 1.45E-08 | 13 |
| BP | GO:0007568 | aging | 11/41 | 9.15E-11 | 3.14E-08 | 1.45E-08 | 11 |
| BP | GO:0003013 | circulatory system process | 13/41 | 9.97E-11 | 3.14E-08 | 1.45E-08 | 13 |
| BP | GO:0006915 | apoptotic process | 21/41 | 1.10E-10 | 3.14E-08 | 1.45E-08 | 21 |
| CC | GO:0005739 | mitochondrion | 17/41 | 4.79E-09 | 1.53E-06 | 8.01E-07 | 17 |
| CC | GO:0043025 | neuronal cell body | 10/41 | 3.73E-08 | 5.95E-06 | 3.12E-06 | 10 |
| CC | GO:0044297 | cell body | 10/41 | 1.37E-07 | 1.46E-05 | 7.67E-06 | 10 |
| CC | GO:0036477 | somatodendritic compartment | 11/41 | 3.01E-07 | 2.40E-05 | 1.26E-05 | 11 |
| CC | GO:0097458 | neuron part | 14/41 | 1.94E-06 | 1.24E-04 | 6.51E-05 | 14 |
| CC | GO:0044429 | mitochondrial part | 11/41 | 5.64E-06 | 0.0003 | 0.000157 | 11 |
| CC | GO:0031966 | mitochondrial membrane | 9/41 | 9.86E-06 | 0.000449 | 0.000236 | 9 |
| CC | GO:0005740 | mitochondrial envelope | 9/41 | 1.48E-05 | 0.000589 | 0.000309 | 9 |
| CC | GO:0099503 | secretory vesicle | 10/41 | 2.29E-05 | 0.000812 | 0.000426 | 10 |
| CC | GO:0044456 | synapse part | 9/41 | 8.35E-05 | 0.002185 | 0.001147 | 9 |
| MF | GO:0098772 | molecular function regulator | 15/40 | 6.57E-07 | 0.000237 | 0.00013 | 15 |
| MF | GO:0043168 | anion binding | 18/40 | 6.65E-06 | 0.001201 | 0.000658 | 18 |
| MF | GO:0030295 | protein kinase activator activity | 4/40 | 1.93E-05 | 0.002327 | 0.001276 | 4 |
| MF | GO:0019209 | kinase activator activity | 4/40 | 2.70E-05 | 0.002441 | 0.001338 | 4 |
| MF | GO:0044877 | protein-containing complex binding | 10/40 | 4.72E-05 | 0.003406 | 0.001867 | 10 |
| MF | GO:0048037 | cofactor binding | 7/40 | 6.50E-05 | 0.003908 | 0.002143 | 7 |
| MF | GO:0005179 | hormone activity | 4/40 | 9.89E-05 | 0.004723 | 0.002589 | 4 |
| MF | GO:0000166 | nucleotide binding | 14/40 | 0.00012 | 0.004723 | 0.002589 | 14 |
| MF | GO:1901265 | nucleoside phosphate binding | 14/40 | 0.00012 | 0.004723 | 0.002589 | 14 |
| MF | GO:0016491 | oxidoreductase activity | 8/40 | 0.000131 | 0.004723 | 0.002589 | 8 |
