# Supplementary material for: Exploring hub genes and crucial pathways linked to oxidative stress in bipolar disorder depressive episodes through bioinformatics analysis
Source: Front Psychiatry. 2024 Mar 6;15:1323527. doi: 10.3389/fpsyt.2024.1323527 (PMC10950934; doi:10.3389/fpsyt.2024.1323527)
Supplement: Supplementary file 7 [file Table_7.docx]

**Table S7 KEGG enrichment analysis.**

| ID | Description | GeneRatio | *p-value* | *p-adjust* | *q-value* | Count |
| --- | --- | --- | --- | --- | --- | --- |
| hsa05212 | Pancreatic cancer | 6/35 | 7.72E-07 | 0.000134036 | 5.23E-05 | 6 |
| hsa04012 | ErbB signaling pathway | 6/35 | 1.62E-06 | 0.000134036 | 5.23E-05 | 6 |
| hsa04912 | GnRH signaling pathway | 6/35 | 2.76E-06 | 0.000134036 | 5.23E-05 | 6 |
| hsa05166 | Human T-cell leukemia virus 1 infection | 8/35 | 3.74E-06 | 0.000134036 | 5.23E-05 | 8 |
| hsa04933 | AGE-RAGE signaling pathway in diabetic complications | 6/35 | 4.22E-06 | 0.000134036 | 5.23E-05 | 6 |
| hsa04218 | Cellular senescence | 7/35 | 5.06E-06 | 0.000134036 | 5.23E-05 | 7 |
| hsa05161 | Hepatitis B | 7/35 | 5.49E-06 | 0.000134036 | 5.23E-05 | 7 |
| hsa04730 | Long-term depression | 5/35 | 5.76E-06 | 0.000134036 | 5.23E-05 | 5 |
| hsa04668 | TNF signaling pathway | 6/35 | 8.16E-06 | 0.000168651 | 6.59E-05 | 6 |
| hsa04664 | Fc epsilon RI signaling pathway | 5/35 | 1.07E-05 | 0.000194952 | 7.61E-05 | 5 |
